# Understanding the complexity, patterns, and correlates of alcohol and other substance use among young people seeking help for mental ill-health

**DOI:** 10.1007/s00127-023-02444-w

**Published:** 2023-03-13

**Authors:** Caroline X. Gao, Kate M. Filia, Gillinder Bedi, Jana M. Menssink, Ellie Brown, Debra J. Rickwood, Alexandra G. Parker, Sarah E. Hetrick, Helen Herrman, Ian Hickie, Nic Telford, Patrick D. McGorry, Sue M. Cotton

**Affiliations:** 1 Centre for Youth Mental Health, The University of Melbourne, Parkville, VIC Australia; 2 Orygen, 35 Poplar Road, Parkville, VIC Australia; 3 School of Public Health and Preventive Medicine, Monash University, Melbourne, VIC Australia; 4 Headspace, National Youth Mental Health Foundation, Melbourne, VIC Australia; 5 Faculty of Health, University of Canberra, ACT, Canberra, Australia; 6 Institute for Health and Sport, Victoria University, Melbourne, VIC Australia; 7 Department of Psychological Medicine, University of Auckland, Auckland, New Zealand; 8 Brain and Mind Centre, The University of Sydney, Camperdown, NSW Australia

**Keywords:** Alcohol and substance use, Alcohol and drug use, Substance use harms, Youth mental health, Mental ill-health

## Abstract

**Purpose:**

Use of alcohol and other substances is a multifaceted issue impacting young people across multiple life domains. This paper aims to elucidate patterns of substance use and associated demographic and clinical factors among young people seeking treatment for their mental health.

**Methods:**

Young people (12–25 years old) were recruited from five youth-specific primary mental health (“*headspace*”) services in Australia. Self-reported substance use and harms in the past 3 months were measured using WHO-ASSIST. Network analyses were conducted to evaluate interrelationships between use and harms associated with different substances. Subgroups were then identified based on whether participants reported using high centrality substances, and associated demographic and clinical factors were assessed with multinomial logistic regression.

**Results:**

1107 youth participated. 70% reported use of at least one substance in the past 3 months, with around 30% of those reporting related health, social, legal or financial problems. Network analysis highlighted substantial interconnections between use and harm indicators for all substances, with amphetamine-type stimulants (ATS) and cannabis being high central substances. Higher levels of substance use and harms were reported in subgroups with ATS or cannabis use and different risk factors were associated with these subgroups.

**Conclusions:**

Findings highlight the importance of screening for substance use in youth primary mental healthcare settings, offering a key opportunity for early intervention. Clinicians should be aware of the inner connections of use and harms of different drugs and the role of cannabis and amphetamine use as a marker for more substance use profiles.

**Supplementary Information:**

The online version contains supplementary material available at 10.1007/s00127-023-02444-w.

## Introduction

Use of alcohol and other substances is complex and multifaceted, with substance use problems related to many individual, social and environmental factors [1]. Substance use may be particularly problematic during adolescence and early adulthood due to factors such as increased risk-taking behaviours and underdevelopment of executive functions [2]. Early substance use is associated with polysubstance use [3], as well as several long-term harms, including substance use disorders, cognitive difficulties, behavioural issues, interrupted education and social functioning, financial and legal problems, and morbidity and early mortality [4].

Problematic substance use also commonly co-occurs with mental ill-health (broad term referring to mental illness and mental health problems). In the general Australian population, the 2019 National Drug Strategy Household Survey (NDSHS) indicated that people with a mental ill-health were twice as likely to smoke cigarettes daily and 1.7 times as likely to have used any illicit substance in the past year compared to their peers without mental ill-health [5]. This discrepancy may be larger among young people. The 2017 Australian Secondary Students’ Alcohol and Drug Survey (ASSAD) found students with a diagnosis of mental disorder were three to six times more likely to report using illicit drugs compared to those without mental ill-health [6]. Similar trends were also observed in other countries such as the UK and the USA [7, 8]. The link between substance use and mental ill-health is related to shared underlying risk factors (e.g. chronic stress, trauma and adverse childhood experiences) and possible bidirectional causal associations [9–11].

It is critical to understand substance use in youth with mental ill-health given its high prevalence, as well as the negative impact substance use can have on functioning, treatment adherence and the effects of psychotropic medications [12]. Substance use may also contribute to the development and/or worsening of severe mental ill-health such as psychosis and suicide risk [13].

Although substance use is frequently examined in population surveys and cohorts, studies focusing on substance use in young people with emerging mental ill-health are less common [14]. In clinical settings, although substance use might be acknowledged, it may not be seen as serious enough to warrant the attention given limited mental health service resources. However, this represents a significant missed window of opportunity for early identification and intervention for substance use-associated harms in this high-risk population [15]. Hence, there is a need to increase understanding of patterns of substance use and associated risk factors in this population to guide targeted early intervention and treatment approaches.

There is also an opportunity for new methodologies to better capture the nature of substance use in young people. Overall patterns of substance use and associated harms may be difficult to evaluate due to interactions between different substances [16]. Traditionally, latent class analysis has been used to classify individuals into distinct groups [17, 18]. However, this method only evaluates possible subgroups with different substance-using patterns and does not reveal independent associations between the use of different substances.

As an alternative, network analysis allows the evaluation of direct and indirect associations among factors [19, 20]. This technique is increasingly applied, however, to our knowledge, only one study by Rhemtulla and colleagues [21] has adopted this method, albeit in an adult population (to evaluate associations between symptoms of substance use disorder for an individual substance but not between substance types). Network analysis is particularly useful in evaluating complex systems to gain an understanding of influential factors within the system.

Here, we applied network analysis to explore links between use and harms (e.g. health, social, legal or financial problems) associated with different types of substance use in young people seeking help for mental ill-health. The specific aims of the study were to: (i) report prevalence and patterns of substance use in a large cohort of young people seeking primary mental health care for emerging mental ill-health; (ii) examine interrelationships between the use and harms associated with different substances; (iii) explore demographic and clinical correlates associated with the use of substances that plays a more central role among all substances.

## Method

### Design and participants

This analysis is a part of a larger project focused on developing better outcomes measures for young people with mental ill-health, see details elsewhere [22]. In brief, young people (12–25 years old) were recruited from five youth-focused primary mental health clinics (*headspace* services) in Australia between September 2016 and April 2018 (three metropolitan and two regional centres). *headspace* is a unique primary healthcare setting developed in Australia to provide accessible, evidence-based, youth-friendly, and integrated care for mental health problems in young people aged 12–25 years [23]. All young people presenting for the first time with mental health and/or substance use issues were eligible to participate. The study questionnaire included a range of self-report demographic, clinical and functioning measures assessed both at baseline and 3-month follow-up [22]. Here we report only on baseline data and measures pertinent to this analysis.

### Measures

Substance use and associated harms were assessed using the World Health Organization Alcohol, Smoking, and Substance Involvement Screening Test-Version 3, WHO-ASSIST 3.0 [24]. This measures the frequency of use (past 3 months and lifetime) of substances in ten categories: tobacco products, alcoholic beverages, cannabis, cocaine, amphetamine-type stimulants (ATS), inhalants, sedatives or sleeping pills, hallucinogens, opioids, and other substances) and five associated harm indicators (see Table S1 in Supplementary Material). The total summed score for each substance category represents the past 3-month risk of harmful use [24]. The frequency of substance use over the past 3 months represented participants’ recent use patterns.

Primary diagnoses based on the Diagnostic and Statistical Manual of Mental Disorders fifth edition (DSM-5) [25] were obtained from participants’ medical records. Due to the early intervention nature of *headspace* services, sub-threshold diagnoses were also included. The Patient Health Questionnaire (PHQ-9) [26] was used to measure depressed mood and somatic symptoms related to depression over the past 2 weeks. Generalised anxiety symptoms during the past 2 weeks were measured with the Generalised Anxiety Disorder (GAD-7) scale [27]. Four other clinical symptom measurements which may be associated with different substance use profiles were also evaluated, including the ten-item Rumination Response Scale (RRS-10; rumination) [28], the Pittsburgh Sleep Quality Index (PSQI; sleep quality and disturbances) [29], the Clinical Anger Scale (CAS; anger) [30], and the Prodromal Questionnaire–16 (PQ-16; psychosis risk) [31].

### Procedure

Informed consent was obtained from the young person and a parent/guardian for those aged < 18 years. Following consent, participants completed a comprehensive questionnaire (via tablet) under the supervision of a research assistant. A medical file review by research assistants provided additional clinical information such as diagnosis.

### Statistical analysis

Analyses were conducted using **R** version 4.0.2 (2020-06-22) and versions of packages used are provided in Table S2. Detailed statistical procedures and statistical packages and functions used are provided in Supplementary Material and a brief summary is included below.

#### Evaluation of prevalence of use and harm

Prevalence of different types of substance use, polysubstance use and associated harms in the past 3 months were first examined by demographic subgroups.

#### A zero-order correlation network

A zero-order correlation network analysis was conducted to understand overlaps between all substance use and harm indicators. Multidimensional Scaling (MDS) network plot was used to visualise pairwise tetrachoric correlations (${r}_{\mathrm{t}}$) or the presence of use/harms of individual drugs (binary variable). This plot has a direct graphical interpretation, with the shorter distance representing a stronger association, thereby providing an overview of possible clusters and overall connectivity between the variables (represented by nodes on the network) [20].

#### Partial correlation network and centrality measures

To account for both direct and indirect links between the use of different substances, the Gaussian graphical models (un-regularised using the Glasso algorithm and stepwise model) was used to estimate the partial correlation (${r}_{\mathrm{p}}$) network of ASSIST substance-specific risk score (sum score for each substance log-transformed as nodes) [19]. The centrality of different substances was measured using local centrality indicators (strength, expected influence) and global centrality indicators (betweenness, and closeness) of the nodes on the network [32]. In our analysis, the local centrality measurements represent the strength of overall direct associations of a particular substance on the use and harms of other substances, whereas global centrality measurements identify substances with higher bridging effect (connecting nodes between sub-clusters).

#### Variables associated with high centrality substances

We further classified the cohort into subgroups based on whether the participants used high centrality substances. Multinomial multivariable logistic regression models were used to evaluate which demographic and clinical factors were associated with different types of substance use. Missing data (around 5%) were addressed using multiple imputation [33]. Multiple comparison adjustments were not conducted to avoid increases in type II error rate [34] for this exploratory study.

## Results

The cohort of 1107 young people has been described elsewhere [22]. The median age of participants was 18 years (IQR 16, 20), and 65% were females. Approximately, a third of participants reported LGBTIQA+ (lesbian, gay, bisexual, transgender, intersex, queer/questioning) status. Over three-quarters of participants presented with a primary diagnosis of anxiety and/or depression, and only 2% had a primary diagnosis of substance use disorder.

### Substance use patterns

Overall, 70% of participants reported some substance use in the previous 3 months (see Table 1), with 63% reportedly consuming alcohol, 33% smoking tobacco and 31% using an illicit substance(s). Rates of any substance use were almost doubled in 18–25 years old compared with 12–17 years old. Overall rates of substance use were comparable between males and females (sex at birth) with males slightly more likely to engage in polysubstance use (use of more than one substance in past 3 months). Participants of LGBTIQA+ status reported higher rates of alcohol and illicit substance use. Harms associated with substance use were also common. About half of the cohort reported urges to use at least one substance in the past 3 months, and about one-fifth or more reported health, social, legal or financial problems, failure to meet normal expectations, or concerns from close others about their substance use.

**Table 1:** Past 3-month substance use profile of 1107 young people presenting for mental health care by age group and sex

Characteristic	Overall (*N* = 1107)	Age			Sex at birth			LGBTIQA+ status		
		12–17 (*n* = 500)	18–25 (*n* = 607)	*p* value	Female (*n* = 717)	Male (*n* = 389)	*p* value	No (*n* = 738)	Yes (*n* = 301)	*p* value
Tobacco use	351 (33%)	100 (21%)	251 (43%)	< 0.001	218 (32%)	133 (36%)	0.221	232 (32%)	109 (37%)	0.174
Alcohol use	665 (63%)	184 (39%)	481 (82%)	< 0.001	452 (66%)	212 (57%)	0.004	444 (62%)	206 (69%)	0.029
Illicit substance use^a^	324 (31%)	90 (19%)	234 (40%)	< 0.001	203 (30%)	120 (32%)	0.406	207 (29%)	108 (36%)	0.020
Other substance use^b^	140 (13%)	40 (8.5%)	100 (17%)	< 0.001	93 (14%)	46 (12%)	0.638	89 (12%)	49 (17%)	0.095
Polysubstance use^c^				< 0.001			0.015			0.053
No drug use	320 (30%)	252 (53%)	68 (11%)		191 (28%)	129 (34%)		227 (31%)	73 (24%)	
Using one drug only	298 (28%)	98 (21%)	200 (34%)		211 (31%)	87 (23%)		204 (28%)	83 (28%)	
Polydrug use	449 (42%)	125 (26%)	324 (55%)		288 (42%)	160 (43%)		296 (41%)	143 (48%)	
Polysubstance use (excluding tobacco and alcohol)^d^				< 0.001			0.420			0.043
No drug use	676 (64%)	360 (76%)	316 (54%)		443 (64%)	233 (62%)		475 (66%)	171 (57%)	
Using one drug only	240 (23%)	81 (17%)	159 (27%)		158 (23%)	82 (22%)		152 (21%)	79 (27%)	
Polydrug use	146 (14%)	32 (6.8%)	114 (19%)		87 (13%)	58 (16%)		96 (13%)	48 (16%)	
Harms associated with any drug use										
Urge to use	559 (52%)	149 (31%)	410 (69%)	< 0.001	374 (54%)	185 (49%)	0.134	369 (51%)	174 (58%)	0.036
Led to problems^e^	242 (23%)	52 (11%)	190 (32%)	< 0.001	141 (20%)	101 (27%)	0.021	166 (23%)	74 (25%)	0.564
Failed normal expectations	205 (19%)	54 (11%)	151 (26%)	< 0.001	130 (19%)	75 (20%)	0.721	133 (18%)	68 (23%)	0.122
Caused concerns	240 (22%)	67 (14%)	173 (29%)	< 0.001	139 (20%)	101 (27%)	0.015	162 (22%)	75 (25%)	0.376

Statistics presented are n (%) with *p* value estimated from Chi-squared test. Missing data include 1 for sex at birth, 68 for LGBTIQA+ , 52 for tobacco use, 48 for alcohol use, 45 for illicit drugs use, 50 for other drug use (sedatives, Inhalants or other), 40 for polysubstance use (including tobacco and alcohol), 45 for polysubstance use (excluding tobacco and alcohol), 40 for urge to use, 40 for led to problems, 40 for failed normal expectation and 40 for caused concerns

^a^Illicit substance includes ATS, cannabis, cocaine, hallucinogens and opioids

^b^Other substances include sedatives, inhalants or other substance which is not captured by the main WHO-ASSIST substance groups but was reported as a free text field

^c^Use of more than one substance in the past 3 months including tobacco and alcohol

^d^Use of more than one substance in the past 3 months excluding tobacco and alcohol

^e^Led to health, social, legal or financial problems

### Association networks

**Fig. 1:**
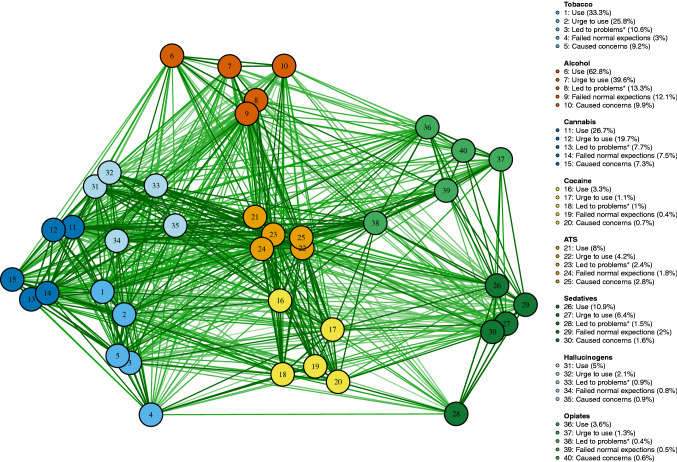
Network plot of zero-order tetrachoric correction between different substance use and harm indicators (dichotomised to “Yes” or “No” from frequency). Links were displayed when there was evidence of pairwise association (*p* < 0.05 using Fisher’s exact test). *Led to health, social, legal or financial problem

**Fig. 2:**
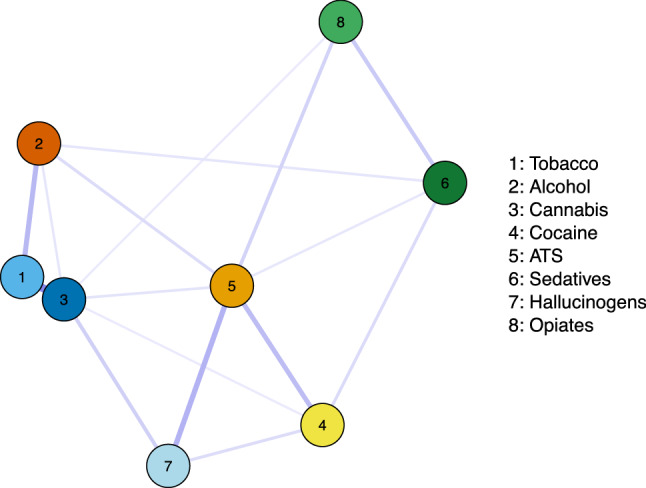
Partial correlation network plot of log-transformed WHO-ASSIST substance-specific risk scores. Line thickness indicates strength of estimated partial correlations

In the zero-order tetrachoric network (see Fig. 1), there were substantial links between use/harms of different substances (mean ${r}_{\mathrm{t}}=0.44$, see Figure S1 in Supplementary Material). Additional sub-clusters were observed, including tobacco, cannabis and hallucinogens; ATS and cocaine. Opiates use/harms were closely linked to use/harms associated with ATS and sedatives. The partial correlation network and associated centrality indicators are plotted in Figs. 2 and S2. After controlling for possible confounding effects between substances, the association between most pairs of substances was retained (all ${r}_{\mathrm{p}}>0$) with strong links between: (i) tobacco and cannabis (${r}_{\mathrm{p}}$ = 0.48); (ii) ATS and hallucinogens (${r}_{p}$ = 0.3); (iii) tobacco and alcohol (${r}_{\mathrm{p}}\hspace{0.17em}$= 0.28); (iv) ATS and cocaine (${r}_{\mathrm{p}}$ = 0.26); (v) and sedatives and opiates (${r}_{\mathrm{p}}$=0.21).

ATS risk score had the highest local (strength and expected influence) and global (betweenness and closeness) centrality on the network, followed by cannabis with high local centrality. The closeness centrality of cannabis use was slightly lower than hallucinogens.

Considering the high centrality role of ATS and cannabis, we divided the total cohort into four groups for further analysis, namely: (i) ATS use (may also use other substances; *n* = 84); (ii) cannabis use without ATS (may also use other substances; *n* = 223); (iii) use of substances other than cannabis and ATS, which was a group with primarily alcohol and/or tobacco use (subsequently referred to as alcohol/tobacco use, *n* = 435); (iv) no recent substance use (*n* = 325). The substance use and harm patterns of these groups, as shown in Tables 2 and S3, suggest clear increases in the number of substances used, frequency of using, and associated harms from the primarily alcohol/tobacco use group to the ATS use group.

**Table 2:** Patterns of substance use of 1107 young people presenting for mental health care by substance use group

Characteristic	Overall (*N* = 1107)	Alcohol/tobacco (*n* = 435)	Cannabis (*n* = 223)	ATS (*n* = 84)	*p* value
Number of substances used	1 (0, 2)	1 (1, 2)	3 (2, 3)	5 (4, 6)	< 0.001
Tobacco use	351 (33%)	123 (29%)	158 (73%)	70 (83%)	< 0.001
Alcohol use	665 (63%)	393 (91%)	191 (87%)	81 (96%)	0.042
Cannabis use	282 (27%)	0 (0%)	223 (100%)	59 (70%)	< 0.001
Cocaine use	35 (3.3%)	6 (1.4%)	8 (3.7%)	21 (25%)	< 0.001
ATS	84 (7.6%)	0 (0%)	0 (0%)	84 (100%)	–
Sedative use	115 (11%)	56 (13%)	29 (13%)	30 (36%)	< 0.001
Hallucinogens use	52 (5.0%)	4 (0.9%)	18 (8.3%)	30 (36%)	< 0.001
Opioids use	38 (3.6%)	8 (1.9%)	12 (5.6%)	18 (21%)	< 0.001
Highest reported frequency of using any substance					< 0.001
Never	302 (31%)	0 (0%)	0 (0%)	0 (0%)	
Once or twice	186 (19%)	157 (39%)	25 (13%)	4 (5.9%)	
Monthly	119 (12%)	87 (22%)	29 (15%)	3 (4.4%)	
Weekly	166 (17%)	92 (23%)	54 (27%)	20 (29%)	
Daily or almost daily	193 (20%)	63 (16%)	89 (45%)	41 (60%)	
Harms associated with any substance use					
Urge to use	559 (52%)	279 (64%)	197 (88%)	81 (96%)	< 0.001
Led to problems^a^	242 (23%)	88 (20%)	100 (45%)	54 (64%)	< 0.001
Failed normal expectations	205 (19%)	74 (17%)	83 (37%)	48 (57%)	< 0.001
Caused concerns	240 (22%)	89 (20%)	93 (42%)	58 (69%)	< 0.001

Statistics presented are median (IQR) and *n* (%) with statistical tests of Kruskal–Wallis test and chi-square test of independence. Participants were grouped according to ATS use (may use other substances), cannabis use without ATS (may use other substances), primarily alcohol and tobacco (use other substance use without cannabis or ATS) and no substance use. Missing data include 40 for substance use group and number of substances used, 52 for tobacco use, 48 for alcohol use, 50 for cannabis use, 57 for cocaine use, 54 for sedative use, 59 for Hallucinogens use, 57 for opioids use, 141 for highest reported frequency of using any substance, 40 for urge of use, 40 for led to problems, 40 for failed normal expectation and 40 for caused concerns

^a^Led to health, social, legal or financial problems

### Associations with demographic and clinical variables

Demographic and clinical variables that characterise the four substance use groups are presented in Table S4. The four groups had varying profiles across demographic factors. Multivariable associations between demographic and clinical measures and the four substance groups, estimated using multinomial logistic regression, are provided in Table 3. Compared with participants who reported no substance use, those who reported substance use were older. The relative risk of ATS use (compared with no substance use) was substantially higher among those presenting to regional centres (RRR_Adj_ = 3.13; 95% CI 1.79–5.48) and among those working only (RRR_Adj_ = 8.41 relative to study only; 95% CI 3.18–22.22). In addition, cannabis use was associated with LBGTIQA + status (RRR_Adj_ = 1.66; 95% CI 1.06–2.59), which was not observed for other groups. Compared to the no substance use group, the cannabis group also had higher levels of clinical anger (RRR_Adj_ = 1.37 per one SD change in CAS; 95% CI 1.08–1.74), and the alcohol/tobacco group had slightly poorer sleep (RRR_Adj_ = 1.28 per one SD change in PSQI; 95% CI 1.02–1.59).

**Table 3:** Multinomial multivariable logistic regression predicting substance use group membership

	Alcohol/tobacco vs. no substance use		Cannabis vs. no substance use		ATS vs. no substance use	
	RRR_Adj_ (95% CI)	*p* value	RRR_Adj_ (95% CI)	*p* value	RRR_Adj_ (95% CI)	*p* value
Age in years	1.36 (1.27–1.46)	< 0.001	1.33 (1.23–1.44)	< 0.001	1.58 (1.42–1.77)	< 0.001
Sex at birth						
Female	Ref		Ref		Ref	
Male	0.67 (0.46–0.98)	0.037	1.00 (0.66–1.51)	0.996	0.87 (0.48–1.57)	0.651
LGBTIQA+						
No	Ref		Ref		Ref	
Yes	1.05 (0.70–1.58)	0.806	1.66 (1.06–2.59)	0.027	0.92 (0.48–1.78)	0.807
Region						
Metro	Ref		Ref		Ref	
Regional	1.96 (1.35–2.83)	< 0.001	1.95 (1.30–2.92)	0.001	3.13 (1.79–5.48)	< 0.001
Education and employment status						
Studying only	Ref		Ref		Ref	
Working only	4.03 (1.99–8.15)	< 0.001	3.46 (1.64–7.33)	0.001	8.41 (3.18–22.22)	< 0.001
Studying and working	3.02 (1.95–4.67)	< 0.001	2.01 (1.22–3.32)	0.007	3.56 (1.57–8.04)	0.002
Not studying or working	1.39 (0.77–2.50)	0.272	1.98 (1.08–3.64)	0.028	3.08 (1.25–7.59)	0.015
Primary diagnosis						
Depression	Ref		Ref		Ref	
Anxiety	0.66 (0.39–1.13)	0.129	0.80 (0.42–1.52)	0.492	0.94 (0.38–2.32)	0.901
Depression and Anxiety	0.88 (0.52–1.48)	0.633	1.44 (0.78–2.63)	0.241	0.97 (0.41–2.27)	0.942
Other	0.71 (0.40–1.23)	0.221	1.36 (0.71–2.60)	0.348	2.09 (0.88–4.99)	0.097
PHQ-9	0.87 (0.65–1.17)	0.358	0.88 (0.63–1.22)	0.444	1.09 (0.69–1.73)	0.708
GAD-7	0.86 (0.66–1.11)	0.235	0.86 (0.65–1.15)	0.323	0.81 (0.53–1.23)	0.314
RRS-10	1.06 (0.84–1.34)	0.607	1.06 (0.82–1.36)	0.656	1.40 (0.97–2.01)	0.071
PSQI	1.28 (1.02–1.59)	0.032	1.05 (0.82–1.34)	0.705	1.10 (0.77–1.57)	0.594
CAS	1.08 (0.86–1.35)	0.514	1.37 (1.08–1.74)	0.009	1.09 (0.79–1.51)	0.582
PQ-16	1.23 (1.00–1.52)	0.049	1.12 (0.89–1.41)	0.334	1.25 (0.90–1.73)	0.177

RRR_**Adj**_: relative risk ratio estimated using multivariate multinomial logistic regression with missing data imputed via MICE. Participants were grouped according to ATS use (may use other substances), cannabis use without ATS (may use other substances), primarily alcohol and/or tobacco (use substances other than cannabis or ATS) and no substance use, and PHQ-9 (The Patient Health Questionnaire), GAD-7 (Generalised Anxiety Disorder), RRS-10 (Rumination Response Scale), PSQI (Pittsburgh Sleep Quality Index), CAS (Clinical Anger Scale) and PQ-16 (Prodromal Questionnaire) total scores were standardised for ease of comparison

Comparisons of both cannabis and ATS with alcohol/tobacco use are provided in Table S5, showing few distinguishing factors. Cannabis use is distinguished from alcohol/tobacco used by being male, LGBTIQA + status, primary diagnosis of depression and anxiety or other (compared to depression), and higher anger. ATS use was differentiated from alcohol/tobacco use by older age and other diagnosis (compared to depression).

## Discussion

Substance use and associated harms are complex phenomena and new statistical methods can help to understand their interrelationships. In this analysis, we used a novel statistical approach—network analysis—to examine interconnections between the use and harms of different substances, as well the topology of the substance-using network, to gain an understanding of substance use profiles in young people first presenting to primary youth mental health services. The results reveal the high frequency and significant interconnections of the use of different substances and their associated harms, and show that ATS and cannabis played more central roles with higher overall associations with use and harms of other substances.

The prevalence of substance use in this cohort was considerably higher than the general population. Findings from the 2019 Australian NDSHS show that only 8% of 14–19 years old and 24% of 20–29 years old have ever smoked cigarettes, whereas, in our sample, rates of tobacco use in the past 3 months for 12–17 years old and 18–25 years old were 21% and 43%, respectively [5, 35]. The prevalence of illicit substance use in the past year was reported as 15.9% for 14–19 years old and 31% for 20–29 years old in the national sample [5], whereas 19% of 12–17 years old and 40% of 18–25 years old in our sample reportedly used an illicit substance in the past 3 months. Past 3-month ATS use (8%) was also much higher compared with national figures (0.9% for 14–19 years old and 2.4% for 20–29 years old in the past year) [9].

While most participants were still at the early stage of mental ill-health [22], the interconnections observed between all substance use and harm indicators were substantial. Polysubstance use has been widely reported in youth populations [36, 37]; however, the interplay between different substances has rarely been evaluated. Recent developments in network analysis allow us to better understand the dynamic interactions among symptoms [38].

Some substance-using sub-clusters were identified in the zero-order substance use and harm correlation network, such as the connection between cannabis with tobacco and hallucinogens as well as the connection between ATS with cocaine, hallucinogens and opiates. These major connections remain strong in the partial correlation network, which indicates that these links were not being confounded by other substance use [39]. Although these links could be associated with common underlying factors, it is also possible that early interventions and treatment for one substance may directly impact other substance use by young people. However, longitudinal data are required to confirm the potential causal associations.

Among all the substance groups evaluated, ATS had the highest centrality followed by cannabis. The high centrality, in this content, indicates stronger and wider independent correlations with other substances. In this cohort, ATS use was a marker of the severe end of substance use with young people in this group frequently using other substances (such as cannabis, cocaine, hallucinogens and opiates) and experiencing social and functional impairment associated with their substance use. Similar findings are also observed from cohorts of young people from the general community [40, 41]. Although there was no strong evidence suggesting clinical symptoms were associated with ATS use when other factors were considered, this could be a result of a lack of statistical power due to the small sample size in this group.

Cannabis use also had an important role in the overall substance use network with strong links, particularly with tobacco and hallucinogens. In our cohort, 69% (114 out of 166) of daily tobacco smokers also used cannabis, which is substantially higher compared to non-daily tobacco smokers (18%). These unequal rates are equivalent to an unadjusted odds ratio of 9.8, which is much higher compared with estimates from the general population [42, 43]. Although the link between cannabis and tobacco use has been well established (e.g. gateway effect of tobacco, common co-administration pattern with forms such as blunts, spliffs or vaping, and shared genetic predispositions and environmental factors) [44, 45], this link seems to be stronger among young people with mental ill-health, warranting further investigation. Co-use, particularly co-administration, of cannabis and tobacco can lead to a greater level of harms [45, 46]. The co-use pattern may also become more significant in recent years with vaping devices becoming more popular and accessible [47, 48]. Targeted interventions, particularly delivered in mental health care settings, are needed.

Interestingly, although the primarily alcohol/tobacco group presented with the lowest level of substance use and harms among all three substance use subgroups, harms such as “lead to problems” and “failed normal expectations” were commonly reported. This indicates the high vulnerability of substance use-related harms in the mental health help-seeking youth.

Considering the central role of ATS and cannabis, we further divided the population into four groups and assessed how demographics and clinical factors were associated with substance-using patterns. Results suggest that substance use groups were most strongly related to non-clinical factors. Living in regional areas was found to be one of the most important predictors of using substances, particularly ATS. At the population level, rates of cannabis and ATS use were only slightly higher in regional areas compared with metropolitan areas [5, 49]. In our cohort, less than 6% of participants from metropolitan areas used ATS compared with more than 15% from regional areas. This substantive difference suggests the need for improved integration of care for substance and mental health, particularly in regional areas of Australia.

Another important issue to note is that substance use was associated with engagement in education and/or employment. Those who were working or both studying and working were found more likely to use substances; this was found consistently in models adjusted for other factors such as age and sex. The relative risk ratio of using ATS relative to no substance use was more than 7 times higher in the working only group compared with the studying only group after controlling for confounding factors such as age. Although this high risk may relate to access to money, it is also possible that substance use, particularly ATS use, was related to exposure through workplaces or early school disengagement in this population [40].

There are some limitations of the study. The prevalence of substance use and harms in this cohort may be biased due to participation bias or response bias [22]. Our sample shared some similarities and differences with headspace samples reported previously, but overall it was largely representative of the patient characteristics [22]. However, care must be taken in interpreting the results. The findings may not generalise to other populations in Australia and other countries. For example, a high proportion (30%) of young people reported an LGBTIQA + status, which, although comparable to 26% in the national *headspace* data [50], may be higher compared with other health service settings. The sample also does not represent young people seeking help, but do not have access to care. However, the analytical framework provided here can be replicated in other datasets. Only cross-sectional data were used to evaluate connections between substance use variables, which limits our ability to understand the directions of the network and longitudinal causal associations between substance use. Cross-sectional data also cannot differentiate between-individual relationships and within-individual relationships [32], which suggests that the observed associations may reflect either substance using preferences between people or temporal variations in drug of choice.

## Clinical implications

We found that at first presentation to youth primary mental health services, rates of comorbid substance use were disproportionally high compared to the general population. Moreover, a substantive subset of young people presenting for care reported frequent use of a range of substances, as well as clinically relevant harms associated with their use. Despite this, only about 2–3% of headspace clients present primarily for alcohol and other substance use [51]. This reveals the need for screening and integrated care for both substance use and mental health to accommodate the diverse needs of young people presented in the primary mental healthcare setting. As this high discrepancy may also be a result of a lack of willingness to disclose to clinicians, methods for substance use screening and assessment in clinical services should be co-designed with young people’s involvement. More integrated care could also leverage the ready presentation of young people with substance use for mental health care to facilitate early intervention for substance use problems. Clinicians should also be aware that cannabis and ATS use could be a marker for more complex and multifaceted substance-using patterns, warranting a focus on targeted treatments for these substances.

## Supplementary Information

Below is the link to the electronic supplementary material.

Supplementary file1 (DOCX 1005 KB)

## Data Availability

The datasets used in the current study are available from the corresponding author on reasonable request.
