# Supplementary material for: Understanding the complexity, patterns, and correlates of alcohol and other substance use among young people seeking help for mental ill-health
Source: Soc Psychiatry Psychiatr Epidemiol. 2023 Mar 13;58(10):1457–67. doi: 10.1007/s00127-023-02444-w (PMC10460308; doi:10.1007/s00127-023-02444-w)
Supplement: Supplementary file 1 — Supplementary file1 (DOCX 1005 KB) [file 127_2023_2444_MOESM1_ESM.docx]

Supplementary Material: Understanding the complexity, patterns, and correlates of alcohol and other substance use among young people seeking help for mental illness

# Statistical methods

## Zero-order correlation network of substance use and harm indicators

As we were interested in the presence of use/harms, the WHO-ASSIST questions (Q2-Q6) were dichotomised (“Yes” or “No”). Responses for Q7 “*failed to control substance use*” were not included due to low rates of participants trying to cut down or stop using substances. Inhalants and other substances were also excluded in this network model due to the low prevalence of use (<2%). The network analysis first involves estimation of pairwise associations between all substance use/harm indicators using tetrachoric correlations ($r_{t}$). A Multidimensional Scaling (MDS) network plot was then used to visualise the correlations ($r_{t}$) in two-dimensional space. This plot has a direct graphical interpretation, with the shorter distance representing a stronger association, and thus provides an overview of possible clusters and overall connectivity between the variables (represented by nodes on the network) [1].

### Partial correlation network and centrality measures

The zero-order correlation network cannot distinguish direct from indirect links between variables. For example, the association between drinking alcohol and cannabis use is often confounded by tobacco smoking [2,3]. Probabilistic graphical models, such as Gaussian graphical models (GGM), provide an alternative solution to model both direct and indirect links between variables using partial correlations [4]. We evaluated the GGM (un-regularised using the glasso algorithm and stepwise model) of ASSIST substance-specific risk score (sum score of Q2-Q7 for each substance) [5,6]. Log-plus-one transformation was applied due to skewed distributions of risk scores. Network stability was evaluated using the correlation stability coefficient using 2500 bootstrap samples (CS-coefficient above 0.25 and preferably above 0.5 for metric stable) [5].

GGM provides not only the estimation of independent associations but also centrality measurements (such as strength, expected influence, betweenness, and closeness) of the nodes on the network [4]. Strength centrality measures local centrality via evaluating all the partial correlations that were associated with a node. Expected influence is similar to the strength centrality except for retention of the sign of the weights to capture different contributions of the negative and positive correlation [7]. Closeness and betweenness measure global centrality (connectivity over the entire graph), with closeness evaluating averaged shortest distances between one node to all other nodes on the network, and betweenness measuring the number of shortest paths passing through a node.

## Variables associated with high centrality substances

After evaluating the association network, we further classified the cohort into subgroups based on whether the participants used high centrality substances. Multinomial multivariate logistic regression models were used to evaluate which demographic and clinical factors were associated with different types of substance use. From these models, we extracted the relative risk ratio (RRR), the confidence 95% confidence interval (95% CI) of the RRR, and *p*-value. ‘No substance use’ served as the reference category for these analyses. Missing data (around 5%) were imputed using Multiple Imputation by Chained Equations (MICE) with five imputed datasets [8]. Regression coefficients were pooled using Rubin’s rule in reporting [9].

# Supplementary figures and tables

Table S1: WHO ASSIST Questions for past three months substance use and associated harm

| **Abbreviation** | **Question number** | **Question** | **Score** |
| --- | --- | --- | --- |
| Frequency | Q2 | Frequency of using the substance | 0 `Never' to 6 'Daily or almost daily' |
| Urge to use | Q3 | Frequency of a strong desire or urge to use the substance | 0 ‘never’ to 6 ‘Daily or almost daily' |
| Lead to problems | Q4 | Frequency of use leading to health, social, legal or financial problems | 0 ‘Never’ to 7 'Daily or almost daily' |
| Failed normal expectation | Q5 | Frequency of failure in doing what was normally expected because of the substance use | 0 ‘Never’ to 8 ‘Daily or almost daily’) |
| Cause concerns | Q6 | Whether a friend or relative or anyone else ever expressed concern about the substance use | 0 ‘No, never’ to 6 ‘Yes, in the past 3 months’ |
| Failed to control | Q7 | Whether the participant tried and failed to control, cut down, or stop of the substance | 0 ‘No, never’ to 6 ‘Yes, in the past 3 months’ |

Table S2: Major R functions and packages applied in the analysis

| Analysis | Function | Package | Citation |
| --- | --- | --- | --- |
| Tetrachoric correlation | tetrachoric | psych | Revelle W. Psych: Procedures for psychological, psychometric, and personality research. 2020. |
| Multidimensional scaling | mds | smacof | Mair P, De Leeuw J, Groenen PJF. Smacof: Multidimensional scaling. 2020. |
| Network group | qgraph | qgraph | Epskamp S, Costantini G, Haslbeck J, Isvoranu A. Qgraph: Graph plotting methods, psychometric data visualization and graphical model estimation. 2020. |
| GGM | ggmModSelect | botnet | Epskamp S. Bootnet: Bootstrap methods for various network estimation routines. 2020. |
| Graph centrality | centrality | qgraph | Epskamp S, Costantini G, Haslbeck J, Isvoranu A. Qgraph: Graph plotting methods, psychometric data visualization and graphical model estimation. 2020. |
| Multinomial logistic regression model | multinom | nnet | Ripley B. Nnet: Feed-forward neural networks and multinomial log-linear models. 2020. |
| MICE | mice | mice | Van Buuren S, Groothuis-Oudshoorn K. Mice: Multivariate imputation by chained equations. 2020. |


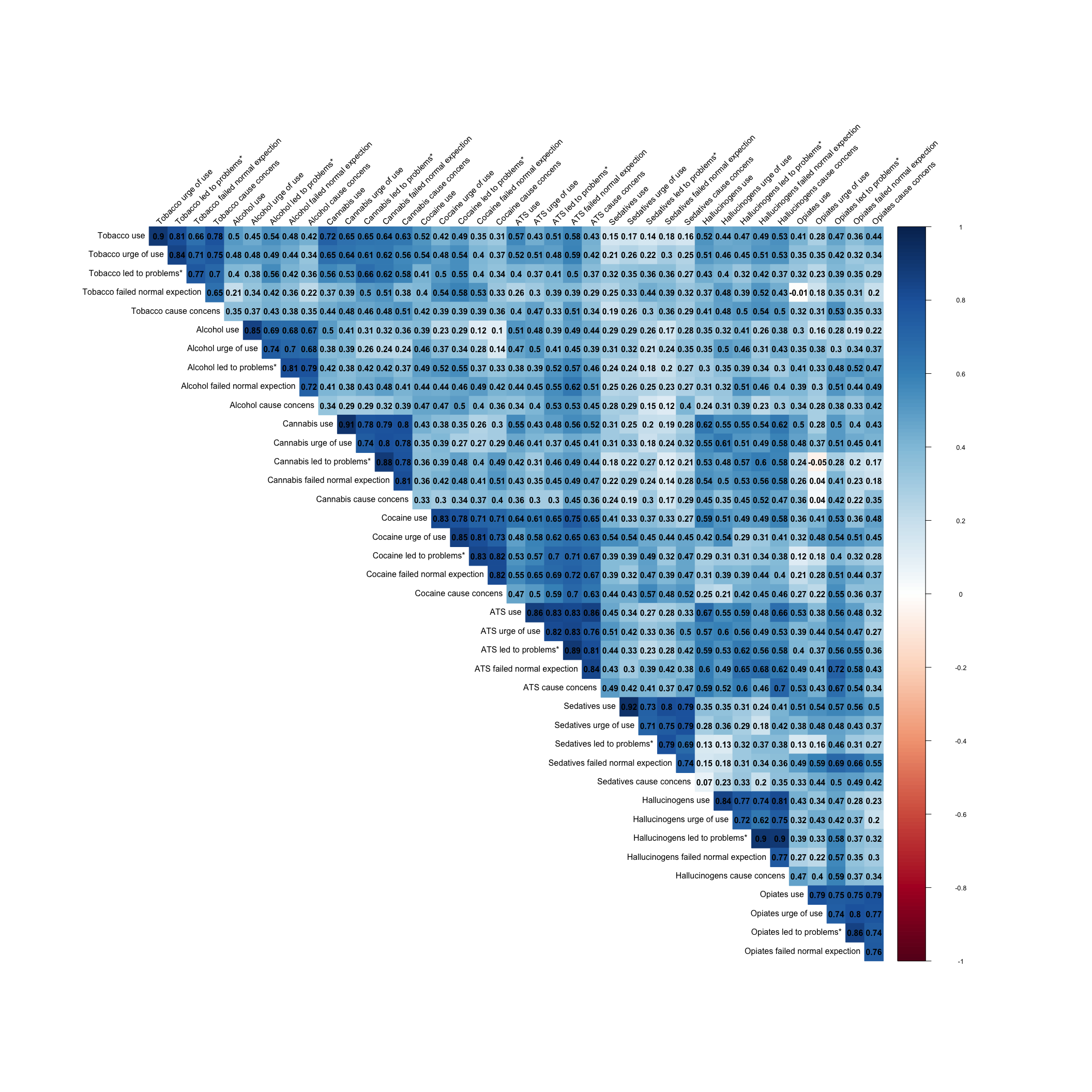


Figure S1: Pairwise tetrachoric correlation between different substance use and harm indicators. * Led to health, social, legal or financial problems


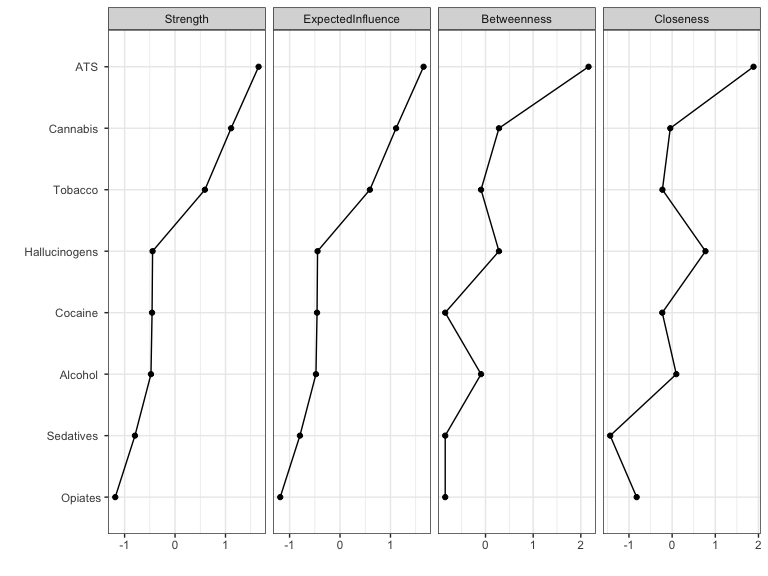


Figure S2: Centrality of the partial correlation log-transformed WHO-ASSIST substance-specific risk scores. Note: bootstrap sampling suggested high level network stability (CS-coefficient>0.5) for strength and expected influence, but slightly lower level of stability with betweenness (CS-coefficient= 0.13) and closeness (CS-coefficient= 0.28). The order of node closeness needs to be interpreted with some care. The betweenness seems to be too sensitive to changes in data, which may cause by lowrates of use of some substances.

Table S3: Frequency of substance use of 1,107 young people participated in the study by substance use group

|  | Overall  (N = 1,107) | Alcohol/tobacco  (N = 435) | Cannabis  (N = 223) | ATS  (N = 84) |
| --- | --- | --- | --- | --- |
| Tobacco |  |  |  |  |
| Never | 704 (67%) | 307 (71%) | 58 (27%) | 14 (17%) |
| Once or twice | 104 (9.9%) | 45 (10%) | 43 (20%) | 16 (19%) |
| Monthly | 32 (3.0%) | 14 (3.3%) | 14 (6.5%) | 4 (4.8%) |
| Weekly | 46 (4.4%) | 16 (3.7%) | 23 (11%) | 7 (8.3%) |
| Daily or almost daily | 169 (16%) | 48 (11%) | 78 (36%) | 43 (51%) |
| Alcohol |  |  |  |  |
| Never | 394 (37%) | 39 (9.0%) | 28 (13%) | 3 (3.6%) |
| Once or twice | 266 (25%) | 182 (42%) | 73 (33%) | 11 (13%) |
| Monthly | 160 (15%) | 103 (24%) | 41 (19%) | 16 (19%) |
| Weekly | 207 (20%) | 92 (21%) | 68 (31%) | 47 (56%) |
| Daily or almost daily | 32 (3.0%) | 16 (3.7%) | 9 (4.1%) | 7 (8.3%) |
| Cannabis |  |  |  |  |
| Never | 775 (73%) | 428 (100%) | 0 (0%) | 25 (30%) |
| Once or twice | 103 (9.7%) | 0 (0%) | 95 (43%) | 8 (9.5%) |
| Monthly | 52 (4.9%) | 0 (0%) | 31 (14%) | 21 (25%) |
| Weekly | 46 (4.4%) | 0 (0%) | 35 (16%) | 11 (13%) |
| Daily or almost daily | 81 (7.7%) | 0 (0%) | 62 (28%) | 19 (23%) |
| Cocaine |  |  |  |  |
| Never | 1,015 (97%) | 420 (99%) | 208 (96%) | 63 (75%) |
| Once or twice | 26 (2.5%) | 4 (0.9%) | 6 (2.8%) | 16 (19%) |
| Monthly | 6 (0.6%) | 1 (0.2%) | 2 (0.9%) | 3 (3.6%) |
| Weekly | 2 (0.2%) | 1 (0.2%) | 0 (0%) | 1 (1.2%) |
| Daily or almost daily | 1 (<0.1%) | 0 (0%) | 0 (0%) | 1 (1.2%) |
| ATS |  |  |  |  |
| Never | 966 (92%) | 426 (100%) | 215 (100%) | 0 (0%) |
| Once or twice | 48 (4.6%) | 0 (0%) | 0 (0%) | 48 (57%) |
| Monthly | 20 (1.9%) | 0 (0%) | 0 (0%) | 20 (24%) |
| Weekly | 13 (1.2%) | 0 (0%) | 0 (0%) | 13 (15%) |
| Daily or almost daily | 3 (0.3%) | 0 (0%) | 0 (0%) | 3 (3.6%) |
| Inhalants |  |  |  |  |
| Never | 1,039 (99%) | 427 (100%) | 210 (97%) | 77 (92%) |
| Once or twice | 12 (1.1%) | 1 (0.2%) | 4 (1.9%) | 7 (8.3%) |
| Monthly | 0 (0%) | 0 (0%) | 0 (0%) | 0 (0%) |
| Weekly | 3 (0.3%) | 1 (0.2%) | 2 (0.9%) | 0 (0%) |
| Daily or almost daily | 0 (0%) | 0 (0%) | 0 (0%) | 0 (0%) |
| Sedatives use |  |  |  |  |
| Never | 938 (89%) | 371 (87%) | 189 (87%) | 54 (64%) |
| Once or twice | 71 (6.7%) | 35 (8.2%) | 17 (7.8%) | 19 (23%) |
| Monthly | 12 (1.1%) | 7 (1.6%) | 3 (1.4%) | 2 (2.4%) |
| Weekly | 18 (1.7%) | 7 (1.6%) | 4 (1.8%) | 7 (8.3%) |
| Daily or almost daily | 14 (1.3%) | 7 (1.6%) | 5 (2.3%) | 2 (2.4%) |
| Hallucinogens |  |  |  |  |
| Never | 996 (95%) | 422 (99%) | 198 (92%) | 53 (64%) |
| Once or twice | 36 (3.4%) | 3 (0.7%) | 16 (7.4%) | 17 (20%) |
| Monthly | 13 (1.2%) | 1 (0.2%) | 2 (0.9%) | 10 (12%) |
| Weekly | 3 (0.3%) | 0 (0%) | 0 (0%) | 3 (3.6%) |
| Daily or almost daily | 0 (0%) | 0 (0%) | 0 (0%) | 0 (0%) |
| Opioids |  |  |  |  |
| Never | 1,012 (96%) | 419 (98%) | 204 (94%) | 66 (79%) |
| Once or twice | 23 (2.2%) | 4 (0.9%) | 6 (2.8%) | 13 (15%) |
| Monthly | 5 (0.5%) | 1 (0.2%) | 3 (1.4%) | 1 (1.2%) |
| Weekly | 8 (0.8%) | 3 (0.7%) | 2 (0.9%) | 3 (3.6%) |
| Daily or almost daily | 2 (0.2%) | 0 (0%) | 1 (0.5%) | 1 (1.2%) |
| Other substances |  |  |  |  |
| Never | 965 (98%) | 398 (98%) | 194 (97%) | 66 (96%) |
| Once or twice | 5 (0.5%) | 2 (0.5%) | 3 (1.5%) | 0 (0%) |
| Monthly | 3 (0.3%) | 1 (0.2%) | 1 (0.5%) | 1 (1.4%) |
| Weekly | 3 (0.3%) | 2 (0.5%) | 1 (0.5%) | 0 (0%) |
| Daily or almost daily | 7 (0.7%) | 4 (1.0%) | 1 (0.5%) | 2 (2.9%) |
| Note: statistics presented are n (%).Participants were grouped according to ATS use (may use other substances), cannabis use without ATS (may use other substances), primarily alcohol and/or tobacco (use substances other than cannabis or ATS) and no substance use. Missing data include 40 for drug_group, 52 for tobacco use in the past 3 months, 48 for alcohol use in the past 3 months, 50 for cannabis use in the past 3 months, 57 for cocaine use in the past 3 months, 57 for amphetamine use in the past 3 months, 53 for inhalants use in the past 3 months, 54 for sedatives use in the past 3 months, 59 for Hallucinogens use in the past 3 months, 57 for opioids use in the past 3 months and 124 for other drugs use in the past 3 months | | | | |

Table S4: Demographics and clinical profile of 1,107 young people presenting for mental health care by substance use group

| **Characteristic** | **Overall**  **(N = 1,107)** | **No substance**  **(n = 325)** | **Alcohol/ tobacco**  **(n= 435)** | **Cannabis**  **(n = 223)** | **ATS**  **(n = 84)** | **p-value** |
| --- | --- | --- | --- | --- | --- | --- |
| **Age in years** | 18 (16, 20) | 15 (14, 17) | 19 (17, 21) | 19 (17, 21) | 20 (19, 22) | <0.001 |
| **Sex at birth** |  |  |  |  |  | 0.015 |
| Female | 717 (65%) | 194 (60%) | 305 (70%) | 137 (61%) | 54 (64%) |  |
| Male | 389 (35%) | 131 (40%) | 129 (30%) | 86 (39%) | 30 (36%) |  |
| **LGBTIQA+** | 301 (29%) | 73 (24%) | 125 (30%) | 79 (37%) | 22 (27%) | 0.019 |
| **Region** |  |  |  |  |  | <0.001 |
| Metro | 707 (64%) | 245 (75%) | 254 (58%) | 132 (59%) | 40 (48%) |  |
| Regional | 400 (36%) | 80 (25%) | 181 (42%) | 91 (41%) | 44 (52%) |  |
| **Education and employment status** | | |  |  |  | <0.001 |
| Studying only | 414 (39%) | 214 (68%) | 112 (27%) | 69 (32%) | 11 (14%) |  |
| Working only | 170 (16%) | 13 (4.1%) | 87 (21%) | 42 (19%) | 26 (32%) |  |
| Studying and working | 306 (29%) | 56 (18%) | 165 (39%) | 59 (27%) | 23 (28%) |  |
| Not studying or working | 159 (15%) | 32 (10%) | 57 (14%) | 48 (22%) | 21 (26%) |  |
| **Primary diagnosis** | |  |  |  |  | <0.001 |
| Depression | 186 (18%) | 46 (15%) | 91 (22%) | 33 (16%) | 13 (16%) |  |
| Anxiety | 271 (26%) | 92 (30%) | 111 (26%) | 42 (20%) | 20 (25%) |  |
| Depression and Anxiety | 344 (33%) | 81 (27%) | 145 (35%) | 81 (39%) | 23 (28%) |  |
| Other | 246 (23%) | 85 (28%) | 73 (17%) | 53 (25%) | 25 (31%) |  |
| **PHQ-9** | 13 (8, 18) | 11 (5, 17) | 13 (9, 18) | 13 (8, 19) | 14 (10, 20) | <0.001 |
| **GAD-7** | 10 (6, 14) | 10 (5, 14) | 11 (7, 14) | 10 (6, 15) | 12 (6, 16) | 0.018 |
| **RRS-10** | 24 (19, 29) | 23 (17, 28) | 24 (20, 29) | 24 (19, 30) | 27 (21, 31) | <0.001 |
| **PSQI** | 8 (5, 11) | 7 (5, 10) | 8 (6, 12) | 8 (6, 11) | 8 (6, 12) | <0.001 |
| **CAS** | 8 (3, 16) | 7 (3, 15) | 7 (3, 16) | 10 (3, 19) | 11 (4, 16) | 0.006 |
| **PQ-16** | 5 (2, 8) | 4 (2, 7) | 5 (2, 8) | 5 (2, 8) | 5 (2, 8) | 0.417 |
| Note: statistics presented are median (IQR) and n (%) with statistical tests of Kruskal-Wallis test and chi-square test of independence. Participants were grouped according to ATS use (may use other substances), cannabis use without ATS (may use other substances), primarily alcohol and/or tobacco (use substances other than cannabis or ATS) and no substance use. Missing data include 40 for substance use group, 1 for sex at birth, 68 for LGBTIQA+, 58 for education and employment status, 60 for primary diagnosis, 39 for PHQ-9, 40 for GAD-7, 41 for RRS-10, 84 for PSQI, 41 for CAS total score and 39 for PQ-16 | | | | | | |

Table S5: Multinomial logistic regression predicting substance use groups among participants who reported any past 3-month substance use

|  | **Cannabis vs Alcohol/tobacco** | | **ATS vs Alcohol/tobacco** | |
| --- | --- | --- | --- | --- |
|  | **RRR_Adj_ (95% CI)** | **p-value** | **RRR_Adj_ (95% CI)** | **p-value** |
| **Age in years** | 0.98 (0.92-1.05) | 0.523 | 1.19 (1.07-1.31) | <0.001 |
| **Sex at birth** |  |  |  |  |
| Female | Ref |  | Ref |  |
| Male | 1.53 (1.05-2.22) | 0.026 | 1.33 (0.77-2.29) | 0.305 |
| **LGBTIQA+** |  |  |  |  |
| No | Ref |  | Ref |  |
| Yes | 1.65 (1.12-2.42) | 0.012 | 0.88 (0.48-1.58) | 0.660 |
| **Region** |  |  |  |  |
| Metro | Ref |  | Ref |  |
| Regional | 1.01 (0.72-1.43) | 0.944 | 1.61 (0.98-2.64) | 0.062 |
| **Education and employment status** | |  |  |  |
| Studying only | Ref |  | Ref |  |
| Working only | 0.83 (0.49-1.41) | 0.496 | 2.11 (0.93-4.79) | 0.075 |
| Studying and working | 0.65 (0.42-1.01) | 0.056 | 1.15 (0.53-2.53) | 0.721 |
| Not studying or working | 1.37 (0.81-2.34) | 0.240 | 2.23 (0.96-5.19) | 0.064 |
| **Primary diagnosis** |  |  |  |  |
| Depression | Ref |  | Ref |  |
| Anxiety | 1.28 (0.72-2.25) | 0.402 | 1.51 (0.66-3.44) | 0.328 |
| Depression and Anxiety | 1.81 (1.10-3.00) | 0.021 | 1.15 (0.53-2.51) | 0.719 |
| Other | 2.00 (1.14-3.53) | 0.017 | 3.27 (1.45-7.34) | 0.004 |
| **PHQ-9** | 1.01 (0.75-1.36) | 0.957 | 1.30 (0.85-1.99) | 0.227 |
| **GAD-7** | 1.01 (0.77-1.31) | 0.964 | 0.91 (0.61-1.35) | 0.631 |
| **RRS-10** | 1.00 (0.80-1.25) | 0.968 | 1.31 (0.94-1.83) | 0.115 |
| **PSQI** | 0.82 (0.66-1.02) | 0.082 | 0.87 (0.64-1.19) | 0.394 |
| **CAS** | 1.32 (1.07-1.62) | 0.010 | 1.08 (0.80-1.46) | 0.602 |
| **PQ-16** | 0.89 (0.72-1.09) | 0.250 | 0.99 (0.73-1.33) | 0.929 |
| Note: RRR**_Adj_** : relative risk ratio estimated using multivariate multinomial logistic regression with missing data imputed via MICE. Participants were grouped according to ATS use (may use other substances), cannabis use without ATS (may use other substances), primarily alcohol and tobacco (use other substance use without cannabis or ATS) and no substance use. The no substance use group were excluded from the multinomial logistic regression and PHQ-9, GAD-7, RRS-10, CAS and PQ-16 total scores were standardised for easy comparison | | | | |

# References

1. Jones PJ, Mair P, McNally RJ (2018) Visualizing Psychological Networks: A Tutorial in R. Frontiers in Psychology 9 (1742). <https://doi.org/10.3389/fpsyg.2018.01742>

2. Hindocha C, Shaban NDC, Freeman TP, Das RK, Gale G, Schafer G, Falconer CJ, Morgan CJA, Curran HV (2015) Associations between cigarette smoking and cannabis dependence: A longitudinal study of young cannabis users in the United Kingdom. Drug and Alcohol Dependence 148:165-171. <https://doi.org/10.1016/j.drugalcdep.2015.01.004>

3. Rioux C, Castellanos-Ryan N, Parent S, Vitaro F, Tremblay RE, Séguin JR (2018) Age of Cannabis Use Onset and Adult Drug Abuse Symptoms: A Prospective Study of Common Risk Factors and Indirect Effects. The Canadian Journal of Psychiatry 63 (7):457-464. <https://doi.org/10.1177/0706743718760289>

4. Epskamp S, Waldorp LJ, Mõttus R, Borsboom D (2018) The Gaussian Graphical Model in Cross-Sectional and Time-Series Data. Multivariate Behavioral Research 53 (4):453-480. <https://doi.org/10.1080/00273171.2018.1454823>

5. Epskamp S, Borsboom D, Fried EI (2018) Estimating psychological networks and their accuracy: A tutorial paper. Behavior Research Methods 50 (1):195-212. <https://doi.org/10.3758/s13428-017-0862-1>

6. Epskamp S (2020) bootnet: Bootstrap Methods for Various Network Estimation Routines.

7. Robinaugh DJ, Millner AJ, McNally RJ (2016) Identifying highly influential nodes in the complicated grief network. Journal of Abnormal Psychology 125 (6):747-757. <https://doi.org/10.1037/abn0000181>

8. van Buuren S, Groothuis-Oudshoorn K (2020) mice: Multivariate Imputation by Chained Equations.

9. Rubin DB (1987) Multiple imputation for nonresponse in surveys. John Wiley and Sons, New York
